# Effect of *Aslagh* Capsule, a Traditional Compound Herbal Product on Oligomenorrhea in Patients with Polycystic Ovary Syndrome: A Three-Arm, Open-label, Randomized, Controlled Trial

**DOI:** 10.31661/gmj.v8i0.1261

**Published:** 2019-06-02

**Authors:** Maryam Bahman, Homa Hajimehdipoor, Soodabeh Bioos, Fataneh Hashem-Dabaghian, Maryam Afrakhteh, Mojgan Tansaz

**Affiliations:** ^1^Department of Traditional Medicine, School of Traditional Medicine, Shahid Beheshti University of Medical Sciences. Tehran, Iran; ^2^Traditional Medicine and Materia Medica Research Center and Department of Traditional Pharmacy, School of Traditional Medicine, Shahid Beheshti University of Medical Sciences, Tehran, Iran; ^3^Department of Traditional Medicine, School of Traditional Medicine, Tehran University of Medical Sciences, Tehran, Iran; ^4^Research Institute for Islamic and Complementary Medicine, Iran University of Medical Sciences. Tehran, Iran; ^5^Department of Obstetrics and Gynecology, Shohadaye Tajrish Hospital, Shahid Beheshti University of Medical Sciences, Tehran, Iran

**Keywords:** *Aslagh*, Polycystic Ovary Syndrome, Iranian Traditional Medicine, *Vitex*, Oligomenorrheaa

## Abstract

**Background:**

Oligomenorrhea is a common complaint in patients with polycystic ovary syndrome (PCOS). There are some useful medicinal recommendations such as *Aslagh* product (include fennel fruits, carrot seeds, chaste tree fruits) in Iranian traditional medicine for the treatment of oligomenorrhea in PCOS. Hence, the present investigation was designed to compare *Aslagh* capsule with metformin on the oligomenorrhea.

**Materials and Methods:**

One hundred fifty women aged between 18-43 years with oligomenorrhea due to PCOS were randomly divided into *Aslagh*, metformin, and *Aslagh* + metformin groups. The occurrence of menstrual bleeding was considered as the primary outcome. Menstrual cyclicity, duration, and volume of the bleeding were also evaluated.

**Results:**

Occurrence of menstrual bleeding was 87.2% in all patients, with no significant difference between the three groups (P> 0.05). Menstrual cyclicity significantly improved from baseline in *Aslagh* and *Aslagh* + metformin groups (P=0.02). Duration of menstrual bleeding was significantly higher in *Aslagh* group in the first and the second menstrual bleeding cycle compared to the other two groups (P<0.05). No significant change was observed in the volume of the bleeding after the intervention in any of the three groups. The occurrence of menstrual bleeding in *Aslagh* group was significantly (P=0.03) higher than the other two groups in the fourth month (drug-free period).

**Conclusion:**

*Aslagh* capsule showed beneficial effects similar to metformin in the treatment of oligomenorrhea in PCOS women and could be suggested for use as an alternative treatment in these patients.

## Introduction

Polycystic ovary syndrome (PCOS) is considered as an endocrine disorder, which affects women during the reproductive age and has an incidence of 5–10% [1]. It is commonly characterized by polycystic ovaries, chronic anovulation, and hyperandrogenism, irregular menstrual cycles, infertility, hirsutism, and acne [1-4]. About 50% of women with PCOS have oligomenorrhoea, and approximately 20% have amenorrhea [5, 6]. Menstrual irregularity in PCOS is associated with impaired insulin sensitivity, type-2 diabetes mellitus, cardiovascular diseases, chronic anovulation, high risk of endometrial hyperplasia, and carcinoma [7-10]. Improvement in menstrual cyclicity may reduce some of these complications [10, 11]. Primary care management typically includes oral contraceptives and insulin-sensitizing agents. Metformin is one of the best treatments in the therapy for reproductive and metabolic disorders caused by PCOS [12, 13]. Each of these therapies has been associated with adverse effects, for example, oral contraceptives may increase the risk of cardiovascular diseases, and metformin may cause gastrointestinal side effects [1, 14-16]; therefore, it is necessary to find some new drugs that could be targeted to treat the disease. In Iranian traditional medicine (ITM), no disease could be attributed to PCOS, but the symptoms of PCOS have been mentioned in different parts of ITM textbooks such as *“Canon of medicine.” “Al-Qanon fi Al-Tibb*” or *“Canon of medicine*” is the medical masterpiece of *Ibn –Sina* or Avicenna (980 – 1037 A.D). Different kinds of uterine diseases have been discussed in the 21st chapter of the book. In this section, amenorrhea and oligomenorrhea are described under the title of Ehtebas Tams, which means menstruation stop or reduction in its duration and amount of flow [17, 18]. One of the herbal products used for the treatment of PCOS symptoms [19-21] is *Aslagh* capsule. *Aslagh* capsule also known as *Raha* capsule is made of *Vitexagnus-castus* L. (Verbenaceae), *Foeniculumvulgare* Mill. (Apiaceae), and *Daucuscarota* (Apiaceae). It is used for the management of oligomenorrhea and amenorrhea in ITM clinics [18, 22].

*Vitex* is a common herbal treatment, used for female reproductive conditions [23]. It is effective in premenstrual syndrome [24], improvement of irregularities of the menstrual cycle [23, 25, 26], corpus luteum formation, infertility [23], and hyperprolactinemia [3].Fennel is a well-known plant with a mild estrogenic effect [27]. It is used in resolving primary dysmenorrhea [28-30], as a spasmolytic agent [31] and also for inducing menstrual bleeding in women with amenorrhea and oligomenorrhea in folk medicine [27]. Carrot is a very popular vegetable used in usual diet worldwide. It is rich in beta-carotene and anthocyanin, which contributes to many health benefits [32]. Carrot has hepatoprotective [33, 34] and antioxidant activities [35, 36]. Various parts of the plant have also been used in folk medicine; e.g., carrot seeds have been used as diuretic and carminative and for stimulation of menstruation [37]. According to the beneficial effects of *Aslagh* capsule ingredients in menstruation and its usage for treatment of oligomenorrhea in ITM, we decided to examine its effect on oligomenorrhea in PCOS patients.

## Materials and Methods

### Patients

The present study was a randomized clinical trial with three groups. According to the pilot study data (type 1 error [0.05] and study power [80%]), the percentage of occurrence of menstruation was considered 90% in *Aslagh* group and 60% in metformin group, thus the sample size was 33 in each group (by the formula of comparing the two proportions) and by adding the study loss (20%) was 41 in each group.

$$n=\frac{2(\bar{p})(1-\bar{p}){\left(Z_{\beta }+Z_{\alpha /2}\right)}^{2}}{{\left(p_{1}-p_{2}\right)}^{2}}$$

One hundred and fifty patients aged between 18-43 years with oligomenorrhea due to PCOS (based on the Rotterdam criteria) [38] who referred the gynecologist in Tehran and Qom, Iran, were enrolled in the study during December 2014 to March 2016. Menstrual cycle length greater than 35 days was considered as Oligomenorrhea [3]. Patients diagnosed with concomitant hyperprolactinemia, hypothyroidism, renal or adrenal insufficiency, diabetes mellitus and a history of drug use for PCOS in the past three months, were not included. Women with suspected pregnancy, planning to have a child in the next three months, breastfeeding with an infant younger than six months and known sensitivity to some drugs or medical plants (especially Apiaceae family) were excluded.

### Recruitment and Randomization

By approval of the ethics and research committees of Shahid Beheshti University of Medical Sciences (approval code: SBMU.REC.1394.81) and registered in the Iranian registry of clinical trial (code: IRCT2015042521937N1), 150 patients were enrolled in the study by informed consent. Researcher enrolled participants and assigned them to interventions. Then, the patients were randomly assigned into three groups (*Aslagh*, metformin, and *Aslagh* + metformin) using block randomization with a block size of 6 and an allocation ratio of 1:1:1. The random allocation sequence was generated by a nurse.

### Preparation of the Drug

Ingredients of *Aslagh* capsule (*V. agnus-castus* L., F. *vulgare* Mill. and D. carota) were provided from a local market (Tehran, Iran). After identification, verification and performing of quality control tests on the materials in Traditional Medicine and Material Medical Research Center, Shahid Beheshti University of Medical Sciences laboratories, they were powdered and mixed in the ratio of 1: 1: 1. The capsules were standardized based on total essential oil content and filled in capsule shells (500 mg) [22]. Metformin 500 mg tablets were obtained from an Iranian Pharmaceutical company (Aria Co, Tehran).

### Intervention

Patients were randomized into three groups. Group 1 received *Aslagh*, 4 capsules daily divided into 2 doses, in the morning on an empty stomach and at night before bedtime, except during the menstrual cycle; group 2 received metformin, 1 tablet three times a day (TDS) after meals; group 3 received *Aslagh*, 4 capsules daily divided into 2 doses, plus metformin 1 tablet TDS. All groups received the treatments for three months and were followed up in the 4th month (drug-free time) for the occurrence of menstrual bleeding in each month. For all patients in the metformin group, the initial dose was 500 mg after dinner for at least one week, and gradually it increased to a final dose of 500 mg TDS to reduce the incidence and severity of gastrointestinal side effects. Patients were advised to use barrier contraception. The subjects did not use other PCOS managements and were asked to keep to their usual diet and lifestyle during the study. All subjects were free to withdraw at any time during the study.

### Outcomes

The menstrual bleeding was considered as the primary outcome. Also, secondary outcomes were the volume and duration of menstrual bleeding. Presence of three times menstruation during the intervention period was considered as regular menses. Menstrual cyclicity (number of cycles/month) was calculated by dividing 30 to the days between two menstrual bleedings [39, 40]. Menstrual cyclicity was compared in the intervening months, and between the baseline and the third month in each group. Considering the significant difference in body mass index (BMI) and waist-hip ratio (WHR) between the three groups at the beginning of the study, analysis of covariance (ANCOVA) test was used to control their confounding effect. The Higham chart (Pictorial blood loss assessment chart, PBAC) was used to determine the volume of menstrual bleedings, which evaluates the menstrual blood loss [41].

### Data Collection

At the beginning of the intervention, each patient was examined clinically, and the researcher recorded their demographic, menstrual and medical histories. Each participant received a drug pack accompanied by the form including the Higham chart. Patients were followed by phone calls every two weeks about their menstruation. Higham charts and the remaining capsules and pills were given in monthly visits. Patients’ compliance was evaluated with a pill count in each visit by the researcher. If more than 20% of the total prescribed dose was missed, the patient was excluded from the study. In case of occurrence of any major side effect, the participants were asked to stop taking the drugs and contact the researcher. The side effects were evaluated based on the self- report symptoms and also a checklist. At the end of the intervention, the patients were asked to express their satisfaction with the medication in a 10-point visual analog scale scoring from 1-10. Patients in group 3 were told to score each of the two drugs separately. Then, a comparison was carried out between *Aslagh* and metformin scores.

### Statistical Analysis

The analysis was done using the SPSS software (Version: 16; IBM, NY, USA). Qualitative variables were presented as number (%) and compared among the three groups using the Chi-square test. Quantitative variables were expressed as the mean ± standard deviation (SD) and were compared among the groups using one-way ANOVA or Kruskal-Wallis tests. A P<0.05 was considered as statistically significant.

## Results

### Quality Control of Plants and Products

Total ash, loss on drying and total essential oil were found to be 8.5, 4 and 2% for F. *vulgare*, 9.5, 4.9 and 2% for D. carota, and 4.95, 7.45 and 2% for V. agnus-castus, respectively. The obtained data and microbial level of the plants were in agreement with the World Health Organization requirements [42]. The total amount of essential oil was 0.01 ml per capsule.

### Demographic Characteristics

**Figure 1:**
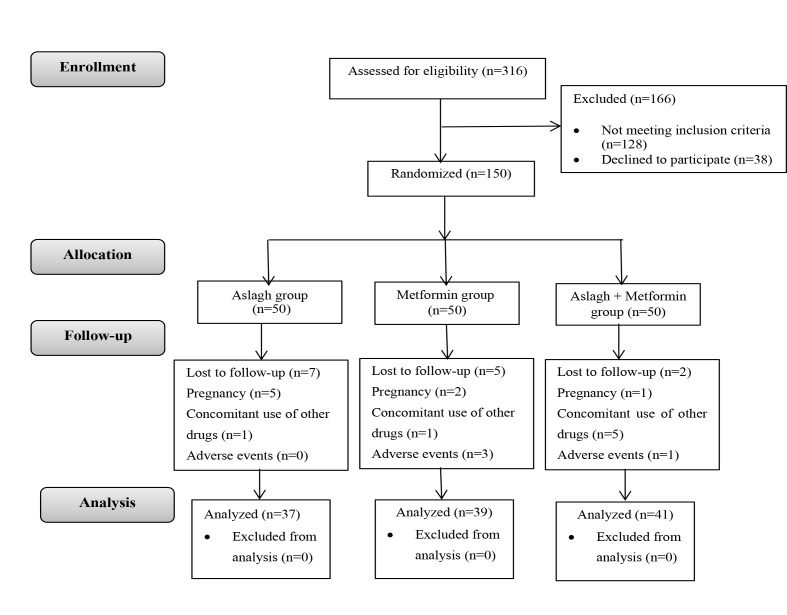
The CONSORT diagram of the study

One hundred and fifty patients were enrolled in the study and were randomly assigned into three groups (Figure-1). The age of the patients ranged from 18 to 43 years. Their mean age was 24.61±5.10 years; their mean age at menarche was 13.23±1.35. ANOVA and chi-square tests showed no significant differences in these variables among the three groups. The demographic data, BMI and WHR are presented in Table-1.

### Menstrual Bleeding

The occurrence of menstrual bleeding was 102 (87.2%) in all patients during the intervention period (three months). This rate was 32 (86.5%) in the *Aslagh* group, 35 (89.7%) in the metformin group, and 35 (85.4%) in the *Aslagh* + metformin group. There was no significant difference between the three groups in the occurrence of menstrual bleeding (P> 0.05). Regular menses occurred in 16 (13.7%) patients. Although the percentage of regular menses was higher in *Aslagh* group (16.2%) compared to metformin (10.3%) and *Aslagh*+ metformin (14.6%), this difference was not significant. ANCOVA used to adjust the confounding parameters (BMI and WHR) that revealed no significant difference between the groups in terms of the number of menstruation during three months (primary outcome) [f (2, 2) =1, P=0.5] and [f (2,3) =2.7, P=0.21], respectively. The occurrence of menstrual bleeding in the groups is presented in Table-2.

### Menstrual Cyclicity Before and During the Intervention

Baseline menstrual cyclicity was 0.43±0.16 in *Aslagh* group, 0.36±0.14 in the metformin group and 0.34±0.13 in *Aslagh*+ metformin group. Menstrual cyclicity increased with treatment in all groups. Menstrual cyclicity was 0.52±0.24 in *Aslagh* group, 0.49±0.23 in the metformin group, and 0.44±0.20 in *Aslagh*+ metformin group in the first cycle. In the second cycle, menstrual cyclicity was 0.83±0.15 in *Aslagh* group, 0.87±0.16 in the metformin group, and 0.80±0.18 in *Aslagh*+ metformin group. In the third cycle, menstrual cyclicity was 0.90±0.13 in *Aslagh* group, 0.89±0.20 in the metformin group and 0.89±0.13 in *Aslagh*+ metformin group. There was no statistically significant difference between the groups in each month.

### Menstrual Cyclicity in the Baseline and Third Month

**Figure 2:**
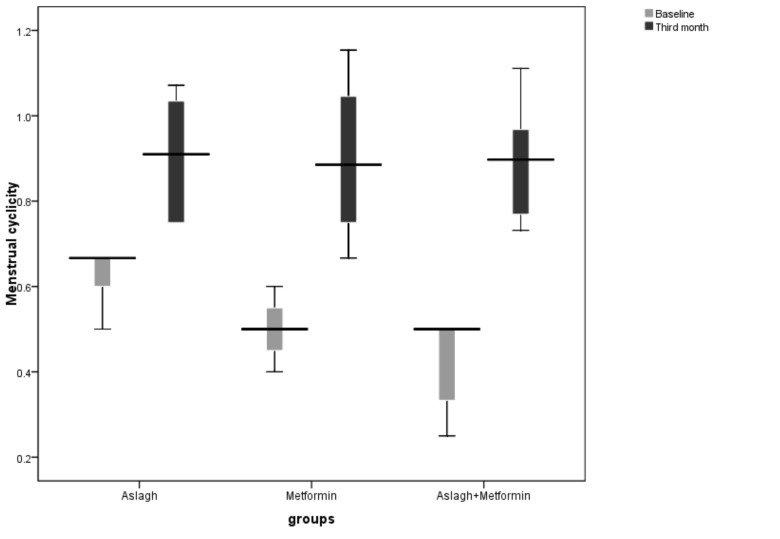
Menstrual cyclicity in the baseline and third month in the three groups

Menstrual cyclicity significantly improved from 0.43±0.16 in baseline to 0.90±0.13 in *Aslagh* group (P=0.02, Paired t-test) and from 0.34±0.13 to 0.89±0.13 in *Aslagh*+ metformin group (P=0.02, Paired t-test). In metformin group, menstrual cyclicity improved from 0.36±0.14 in baseline to 0.89±0.20, but this shift was not statistically significant (P=0.06, Paired t-test). Menstrual cyclicity in the third month and baseline in the three groups are shown in Figure-2.

### Duration and Volume of Menstrual Bleeding

The duration of menstrual bleeding was significantly higher in the *Aslagh* group compared to the other groups in the first (P=0.03) and second (P=0.03) menses after the intervention. Compared to metformin and *Aslagh*+ metformin groups, in the first period, the duration of menstrual bleeding in *Aslagh*+ metformin group was significantly more than metformin group while in the second period the duration of menstrual bleeding in metformin group was more than *Aslagh*+ metformin group,but the difference between the two groups was not significant. The total volume of menstrual bleeding had no significant change in the three months of the intervention in any of the groups ( Table-3).

### Follow-up

The dropped out of the study in the three months was 22% in all patients, 26% in *Aslagh* group compared to 22% in the metformin group and 18% in *Aslagh*+ metformin group,(Chi-Square tests, P=0.6).

After discontinuation of the drug, in the fourth month, the occurrence of menstrual bleeding in *Aslagh* group (56.8%) was significantly higher compared to metformin group (23.1%) and *Aslagh*+ metformin group (Chi-Square test, P= 0.03)

### Side Effects of the Drugs

The most frequent side effects observed, in patients who completed the study (117 patients), included nausea (10 patients in the metformin group, and 13 patients in *Aslagh*+ metformin group) and diarrhea (four patients in the metformin group and one patient in *Aslagh*+ metformin group). The most common side effect of *Aslagh* was rash (two patients in *Aslagh* group), which was not severe and did not result in the discontinuation of the medication.

### Patients Satisfaction of Interventions

Patients’ satisfaction score in the *Aslagh* group was significantly higher compared to metformin (7.69±1.67 in *Aslagh* vs. 6.96±1.94 in metformin, P=0.01).

## Discussion

Oral contraceptives are the first line treatment for menstrual dysfunction in PCOS patients, but most of our patients do not tend to use contraceptives. Actually they referred for treatment of infertility, and the first line of treatment in these patients was metformin in our recruitment center (due to the role of metformin in reducing insulin resistance, restoring menstruation with ovulation, and weight loss in patients that plays an important role in the fertility of these patients); therefore, we considered metformin as control in the study. The results of this study indicated that the menstrual cyclicity increased significantly in the third month compared to the baseline in *Aslagh* and *Aslagh*+metformin groups. The effect of *Aslagh* capsule on the occurrence of menstrual bleeding was equivalent to the effect of metformin. The volume and days of menstrual bleeding increased with *Aslagh* capsule and did not have a significant difference with metformin. The third group did not obtain considerable results. Although we examined the patients’ compliance with a pill count, we guess that due to a large number of medications consumed per day, these patients have not consumed their medication correctly and it was better to use other methods, such as dairy, to record patient compliance.

### Menstrual Bleeding

Thus far, no study has been conducted on the effect of *Aslagh* capsule on oligomenorrhea in patients with PCOS. The present study, however, is similar to the one conducted by Mohebbi-Kian et al. [43] in Hamadan on menstruation. They compared the effect of fennel with low-dose combined oral contraceptive (LD-COC) and placebo in women with amenorrhea due to depo-medroxyprogesterone acetateand showed that women who received LD-COC and fennel were significantly menstruating more than the placebo group [43].

### Menstrual Cyclicity

Menstrual cyclicity increased significantly in the third month compared to the base in *Aslagh* and *Aslagh*+ metformin groups, and this increase was not significant in the metformin group. Considering the significant difference in BMI and WHR, between the three groups in the baseline, these results were analyzed by ANCOVA test, and it was found that BMI and WHR were not confounding factors. Essah et al. (2006) compared the effects of short-term (3-6 months) and long-term (more than six months) metformin on menstrual cyclicity in patients with PCOS [39]. They reported that the number of menstrual cycles was modified from 0.27 per month to 0.60 (in women who took metformin for less than six months) and 0.76 (in women who took metformin for six months and more) [39]. Kort and Lobo study (2014) showed that menstrual cyclicity in patients with PCOS was modified up to 0.75 per month by prescribing cinnamon for six months [40].

### The volume of Menstrual Bleeding

The change of menstrual bleeding volume within three months was not significantly different in any of the groups while in the study conducted by Mohebbi-Kian et al., the volume of menstrual bleeding in fennel group was considerably more than LD-COC and placebo groups [43]. One of the reasons for the difference between our study and Mohebbi-Kian et al. study in menstrual bleeding volume might be the fact that in our study, *Aslagh* capsule was compared with metformin while in their study, fennel was compared with LD-, which reduces menstrual bleeding- and placebo.

### Follow-up

Menstruation occurrence after the discontinuation of medication in patients who took *Aslagh* capsule was more than the other two groups. In the study carried out by Yavari et al. (2015) in Tehran to compare the effect of sesame and progesterone on menstruation in patients with oligomenorrhea, menstruation occurrence in sesame group (4 patients out of 8 patients were followed, 50%) was significantly higher than in progesterone group at the time of drug withdrawal [44]. The total number of patients and the monitored patients in our study were more than that of Yavari et al. study. Our medication is composed of three Emmenagogue drugs whereas they used only sesame.

### Patients Satisfaction of Interventions

In our study, patients’ satisfaction with *Aslagh* treatment was significantly more than with metformin, which could be due to the very low side effects of *Aslagh* compared to metformin. The prevalence of metabolic syndrome in women with PCOS is 43-47%, which is approximately almost twice as much as its prevalence in the normal population of women. High BMI and low high-density lipoprotein are the most frequent components of metabolic syndrome in such patients. The connecting link between PCOS and metabolic syndrome is resistance to insulin. Obesity, dyslipidemia, hypertension, impaired glucose tolerance, high fasting glucose, and cardiovascular disorders are the common metabolic disorders in PCOS [45]. Metabolic syndrome is known to be related to the increased risk of diabetes and atherosclerotic vascular diseases [46]. In ITM, *V. agnus-castus* may reduce swelling of the ovaries and moderate their rigidity [47, 48]; therefore, this plant can be effective in PCOS in which ovaries are big and stiff [10, 49]. It has dopaminergic effects and can be connected to DA2 receptors and can induce an inhibitory effect on prolactin [50, 51]. Since prolactin is a factor for folliculogenesis and oligomenorrhea [3], the decrease of its level can be effective in the treatment of PCOS. Moreover, *Vitex* increases the secretion of melatonin from epiphysis [52]. Melatonin is effective in the maturation of oocytes and ovulation [53, 54]. Since oocyte maturation and ovulation are impaired in such patients [10], *Vitex* can be effective in ovulation in these patients by affecting melatonin. Apigenin in VAC has an inhibitory effect on tumoral cells [55] by inhibiting the incidence of oncogenes [56, 57]. Patients with PCOS are at high risks of endometrial hyperplasia and cancer [10, 21] and breast cancer [58]; so, this herb can be effective in this aspect of PCOS. On the other hand, prescribing progesterone for these patients results in menstrual cycles and the prevention of cancer [21]. Therefore, *Vitex* with its phyto-progesterone property [59] leads to normal menstrual cycles in such patients. In different studies, fennel has affected various aspects of PCOS. In the study conducted by Ozbek et al. (2003) on rats, fennel essence reduced liver enzymes, and its hepatoprotective effect was proved [60]. Furthermore, fennel enhances hepatic synthesis of sex hormone-binding globulin, which is bound to testosterone in the bloodstream and reduces serum levels of free testosterone [61]. Patients with PCOS have lipid profile disorders, and many of their symptoms are due to high androgens. Thus, fennel can be effective in such patients with its hepatoprotective effect and the reduction of androgens [45, 62]. It induces menstruation [43, 47, 60, 63] because it contains phytoestrogens such as isoflavones, flavonoids, and coumestans [64]. Phytoestrogens can bind to estrogen receptors and have agonist-antagonist effects by estrogen [65, 66]. In patients with PCOS, the rate of estrogen is high and is converted to androgens [67], and thus phytoestrogens have antagonist effects by binding with estrogen receptors and can set estrogen performance. Carrot seeds eliminate inflammation [47] and can be effective in large ovaries and multiple follicles in PCOS. Carrot seed is a uterine tonic and can help in pregnancy [17, 47]. Therefore, it can be useful in treating infertility, which is a common complication of PCOS [3, 10, 20]. Carrot seed stimulates sexuality [47]; it has been used as aphrodite and aphrodisiac since many years ago [68]. Studies have shown that carrot extracts inhibit lipid peroxidation and have antioxidant and hepatoprotective effects [33, 69]. Carrot is a rich source of carotenoids, particularly alpha and beta-carotenes with potent anti-cancer properties, which can be used in preventing various kinds of cancer such as breast cancer [70-72]. Moreover, carotenoids reduce the risk of diabetes and insulin resistance and can have a useful effect on PCOS, which is the main cause of the disease, insulin resistance, and compensatory hyperinsulinemia against it [45, 73, 74]. All the three ingredients of *Aslagh* capsule are emmenagogue and can affect PCOS in contributing to menstruation, being hepatoprotective and enhancing blood circulation in the uterus and ovaries [17, 47, 48]. Regarding metformin, it has modified clinical and laboratory symptoms in PCOS in previous studies. Metformin develops and regulates menstrual cycles and removes oligomenorrhea [12, 75], reduces BMI and WHR [75], lowers blood pressure [76], and decreases hyperandrogenism symptoms such as acne and hirsutism [75]. Considering laboratory findings, metformin improves insulin sensitivity and glucose tolerance, modifies liver enzymes [75, 77] and lipid profile [76, 78, 79], reduces serum level of free androgens [75], and modifies risk factors for atherosclerosis and cardiovascular events such as plasminogen activator inhibitor-1 [80], endothelin-1 [13], and c-reactive protein [81]. In our study, the effects of *Aslagh* on the removal of oligomenorrhea were similar to and even better than metformin in some cases. The present study is the first to examine the effect of *Aslagh* capsule on menstruation. The timing of our study was short and longer evaluation time in future studies is recommended. In this study, patients were not adjusted to terms of BMI and WHR. This was another limitation on the study. Although BMI and WHR were not as confusing factors in the statistical analysis, it was better to be adjusted at the beginning of the study. Because of the high sample size and the limited cost of research, we were not able to measure the level of hormones such as estrogen, progesterone, follicle-stimulating hormone, and androgens. In the future studies, we intend to evaluate the effect of the *Aslagh* capsule on other aspects of PCOS such as some hormones.

## Conclusion

In the present study, *Aslagh* capsule showed similar effects compared to metformin in menstruation and more considerable effects in menstrual cyclicity. Co-administration of the two drugs did not show better results; therefore, *Aslagh* capsule can be a good alternative to metformin in treating oligomenorrhea in patients with PCOS. Given the similar effects of metformin and *Aslagh* on menstruation, it is recommended that future studies be planned to investigate the effect of *Aslagh* on other PCOS symptoms and disorders such as glucose tolerance, insulin resistance disorders, androgen levels, symptoms of hyperandrogenism, ovulation, and fertility, as well as its effects on lipid profile, liver tests and their comparison with metformin.

## Acknowledgment

This article was drawn from the Maryam Bahman’s Ph.D. thesis (No. 166 and research project no. 151) from the School of Traditional Medicine, Shahid Beheshti University of Medical Sciences. The authors gratefully acknowledge Mr. Esmaiel Nazem, School of Iranian Traditional Medicine, Tehran University of Medical Sciences for the release of any drug formula, Dr. Ghazaleh Heydarirad, School of Traditional Medicine, Shahid Beheshti University of Medical Sciences and Dr. Majid Asghari, School of Iranian Traditional Medicine, Qom University of Medical Sciences for reading the article and guidance us, and Dr. Maryam Hamzeloo-Moghadam, Department of Traditional Pharmacy, School of Traditional Medicine, Shahid Beheshti University of Medical Sciences, Tehran, Iran for English editing.

## Conflict of Interest

The authors declare that there is no conflict of interest.

**Table 1:** Baseline Characteristics in Patients of Study Groups. Data Are Presented as Mean±SD

**Variables**	***Aslagh***	**Metformin**	***Aslagh*** **+Metformin**	**P-value**
**Age (years)**	23.90±4.95	24.64±4.94	25.30±5.42	0.19
**BMI (kg/m** ^2^ **)**	23.33±4.15	26.92±5.95	27.14±5.35	0.00
**WHR**	0.89±0.05	0.92±0.05	0.90±0.06	0.03
**Age at menarche (years)**	13.22±1.49	13.16±1.20	13.32±1.39	0.97
**Duration of disease (years)**	4.64±3.96	4.07±3.51	5.45±5.05	0.36

**BMI:** Body mass index; **WHR:** Waist-hip ratio

**Table 2:** Comparison of the Groups on Occurrence of Menstrual Bleeding in Three Months of Intervention.Data Are Presented as n (%).

**The occurrence of menstrual bleeding**	***Aslagh***	**Metformin**	***Aslagh*** **+Metformin**	**All patients**
**None**	5 (13.5%)	4 (10.3)	6 (14.6)	15 (12.8%)
**Once time**	7(18.9%)	15 (38.5%)	13 (31.7%)	35 (29.9%)
**Two time**	19 (51.4%)	16 (41%)	16 (39%)	51 (43.6%)
**Three times**	6 (16.2%)	4 (10.3%)	6 (14.6%)	16 (13.7%)
**All patients**	37 (100%)	39 (100%)	41 (100%)	117 (100%)

All comparisons were done using chi-square test, P= 0.66

**Table 3:** Comparison of the Groups on Characteristics of Menstrual Bleeding in the Three Months of Intervention. Data Are Presented as Mean±SD

**Characteristics**	***Aslagh***	**Metformin**	***Aslagh*** **+Metformin**	P-value*
**Duration of menstrual** **bleeding, (days)**	Baseline	7.46±1.38	6.79±1.62	7.04±1.64	0.20
	First	7.31±1.90	6.20±1.60	6.85±1.64	0.03
	Second	7.28±1.62	6.35±2.05	6.13±1.20	0.03
	Third	6.83±1.83	6.25±1.50	6.50±1.04	0.92
	P-value between baseline and third months (Wilcoxon test)	0.31	0.31	0.68	-
**Volume of menstrual** **bleeding,(ml)**	Baseline	53.83±21.03	44.46±18.11	55.07±23.88	0.06
	First	52.25±19.72	44.40±15.10	52.51±23.95	0.16
	Second	44.00±17.20	40.25±17.99	49.95±22.76	0.26
	Third	48.83±14.94	37.00±21.11	57.33±22.80	0.31
	P-value between baseline and third month (Wilcoxon test)	0.34	0.46	0.34	-

* According to Higham chart
